# Supplementary material for: SIV Infection and the HIV Proteins Tat and Nef Induce Senescence in Adipose Tissue and Human Adipose Stem Cells, Resulting in Adipocyte Dysfunction
Source: Cells. 2020 Apr 1;9(4):854. doi: 10.3390/cells9040854 (PMC7226797; doi:10.3390/cells9040854)
Supplement: Supplementary file 1 [file cells-09-00854-s001.pdf]

Supplemental Table S1. Macaques characteristics

Macaque	Sex	Age (years)	Weight (kg)	Years post-infection	Viral load (RNA copies/mL)	CD4 count (cell/µL)
1	M	5,6	7,7	1,0	1,93E+4	ND[1]
2	M	4,6	4,9	0,9	2,61E+4	ND[1]
3	F	5,4	3,2	2,3	1,02E+4	111
4	M	4,8	6,4	1,2	1,45E+5	460
5	M	11,1	15,1			
6	M	11,2	12,8			
7	M	6,1	8,3			

[1] Not determined.

Supplemental Table S2. Sequences of the RT-PCR primers for human ASCs and adipocytes.

Gene	Forward sequence	Reverse sequence
CEBPA	5′gacatcagcgcctacatcg3′	5′ggctgtgctggaacaggt3′
PPARG	5′cagtggggatgtctcataa3′	5′cttttggcatactctgtgat3′
FABP4	5′gctttgccaccaggaaagtg3′	5′atgacgcgattccaccaccag3′
PPIA	5′atgctggacccaacacaaat3′	5′tctttgactttgcaaacacc3′

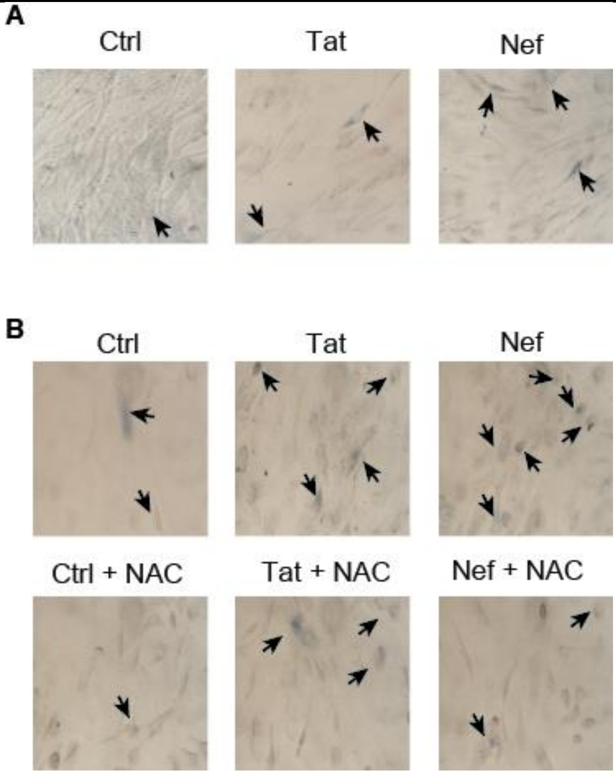

Supplementary Figure S1. The suppression of oxidative stress using NAC prevented Tat- or Nef-induced senescence-associated beta-galactosidase activity in ASCs. ASCs, isolated from different abdominal SCAT healthy donors, were cultured with the HIV proteins Tat or Nef for 15 days. After these 15 days of treatment, we started a concomitant NAC treatment for 10 days. Experiments were performed on day 25 in ASCs, isolated from different subcutaneous abdominal SCAT healthy donors. Senescence was evaluated by SA-β-galactosidase activity at pH 6. Representative micrographs are shown (**A**) after 15 days of HIV-protein treatment and (**B**) after 25 days of HIV-protein treatment with or without NAC treatment.
